# Differences between Lower Extremity Arterial Occlusion vs. Stenosis and Predictors of Successful Endovascular Interventions

**DOI:** 10.3390/medicina59112029

**Published:** 2023-11-17

**Authors:** Damianos G. Kokkinidis, Dimitrios Schizas, Sumant Pargaonkar, Dimitrios Karamanis, Konstantinos S. Mylonas, Natasha Hasemaki, Leonidas Palaiodimos, Dimitrios Varrias, Georgios Tzavellas, Gerasimos Siasos, Christos Klonaris, Amrin Kharawala, David-Dimitris Chlorogiannis, Sotirios Georgopoulos, Christos Bakoyiannis

**Affiliations:** 1 Section of Cardiovascular Medicine, Yale University School of Medicine, New Haven, CT 06510, USA; 2 First Department of Surgery, Laikon General Hospital, School of Medicine, National and Kapodistrian University of Athens, 11527 Athens, Greece; 3 Department of Medicine, Jacobi Medical Center, Albert Einstein College of Medicine, New York, NY 10461, USA; pargaons@nychhc.org (S.P.);; 4 Department of Economics, University of Piraeus, 18534 Piraeus, Greece; 5 Department of Health Informatics, Rutgers School of Health Professions, Newark, NJ 07107, USA; 6 Department of Vascular Surgery, Ball Memorial Hospital, Muncie, IN 47303, USA; 7 3rd Department of Cardiology, Sotiria General Hospital, School of Medicine, National and Kapodistrian University of Athens, 11527 Athens, Greece; 8 Department of Radiology, Brigham and Women’s Hospital, Boston, MA 02115, USA

**Keywords:** peripheral artery disease, claudication, endovascular technique, chronic total occlusion, lower extremity, critical limb-threatening ischemia, atherosclerosis

## Abstract

*Background and Objectives:* In patients with peripheral artery disease, there is insufficient understanding of characteristics that predict successful revascularization of the lower extremity (LE) chronic total occlusions (CTOs) and baseline differences in demographic, clinical, and angiographic characteristics in patients with LE CTO vs. non-CTO. We aim to explore these differences and predictors of successful revascularization among CTO patients. *Materials and Methods:* Two vascular centers enrolled LE-CTO patients who underwent endovascular revascularization. Data on demographics, clinical, angiographic, and interventional characteristics were collected. LE non-CTO arterial stenosis patients were compared. A total of 256 patients with LE revascularization procedures were studied; among them, 120 had CTOs and 136 had LE stenosis but no CTOs. *Results:* Aspirin use (Odds ratio, OR: 3.43; CI 1.32–8.88; *p* = 0.011) was a positive predictor whereas a history of malignancy (OR: 0.27; CI 0.09–0.80; *p* = 0.018) was a negative predictor of successful crossing in the CTO group. The CTO group had a higher history of myocardial infarction (29.2 vs. 18.3%, *p* = 0.05), end-stage renal disease (19.2 vs. 9.6%, *p* = 0.03), and chronic limb-threatening ischemia as the reason for revascularization (64.2 vs. 22.8%, *p* < 0.001). They were more likely to have advanced TransAtlantic Inter-Society Consensus (TASC) stages, multi-vessel revascularization procedures, longer lesions, and urgent treatment. *Conclusions:* The use of aspirin is a positive predictor whereas a history of malignancy is a negative predictor for successful crossing in CTO lesions. Additionally, LE-CTO patients have a higher incidence of comorbidities, which is expected given their higher disease burden. Successful endovascular re-vascularization can be associated with baseline clinical variables.

## 1. Introduction

Over the years, there has been a steady rise in the global prevalence of peripheral artery disease (PAD). The overall global burden of PAD has been estimated to be around 5.6% with a higher prevalence of up to 7.4% among high-income countries [1]. With the increasing prevalence of PAD, there is a higher proportion of patients with complicated vascular disease, including chronic total occlusions (CTO) in the lower extremities (LE). CTO prevalence is as high as 40% among patients with PAD undergoing lower extremity endovascular interventions [2,3]. In the past decade, there has been a shift from surgical revascularization to an endovascular-first approach in complex PAD [4,5,6]. Endovascular approaches in patients with critical limb-threatening ischemia have been shown to be associated with improved amputation-free survival in some studies but they are associated with high rates of reinterventions [4,7,8,9,10,11,12].

CTOs are not limited to peripheral arteries. Coronary and carotid atherosclerosis can also lead to CTOs. Coronary CTOs have been shown to have mainly two distinct histopathological patterns: (i) early coronary CTOs with loose matrix amenable to crossing, and (ii) older coronary CTOs with dense fibrocalcific plaques, which pose difficulty in crossing [13,14,15]. Similarly, relatively more chronic peripheral CTOs can be challenging for vascular interventionalists. Unfortunately, imaging modalities can only help predict the histology of the peripheral arterial CTOs up to a certain degree. As a result, many of the lesions that are attempted with an endovascular-first approach are difficult to cross [16,17]. New techniques and devices have emerged in recent years to overcome the difficulty of crossing these complex lesions [15,16,17,18,19,20,21,22]. Despite these advances in management, failure rates in crossing and successful revascularization in these patients with Infrainguinal CTO remain as high as 30% [23].

Little is known about the baseline differences between patients with peripheral LE-CTO and those without LE-CTO stenotic lesions. Additionally, it is unclear which variables predict the successful endovascular intervention in LE-CTO procedures, even if prior studies have tried to explore this. In the present study, we aim to better understand the baseline characteristic differences that might predispose PAD patients to develop CTOs. Furthermore, identifying the predictors of a successful endovascular intervention in this subset of PAD patients could help select the most appropriate patient subgroup and technique, and avoid complications.

## 2. Materials and Methods

**Figure 1:**
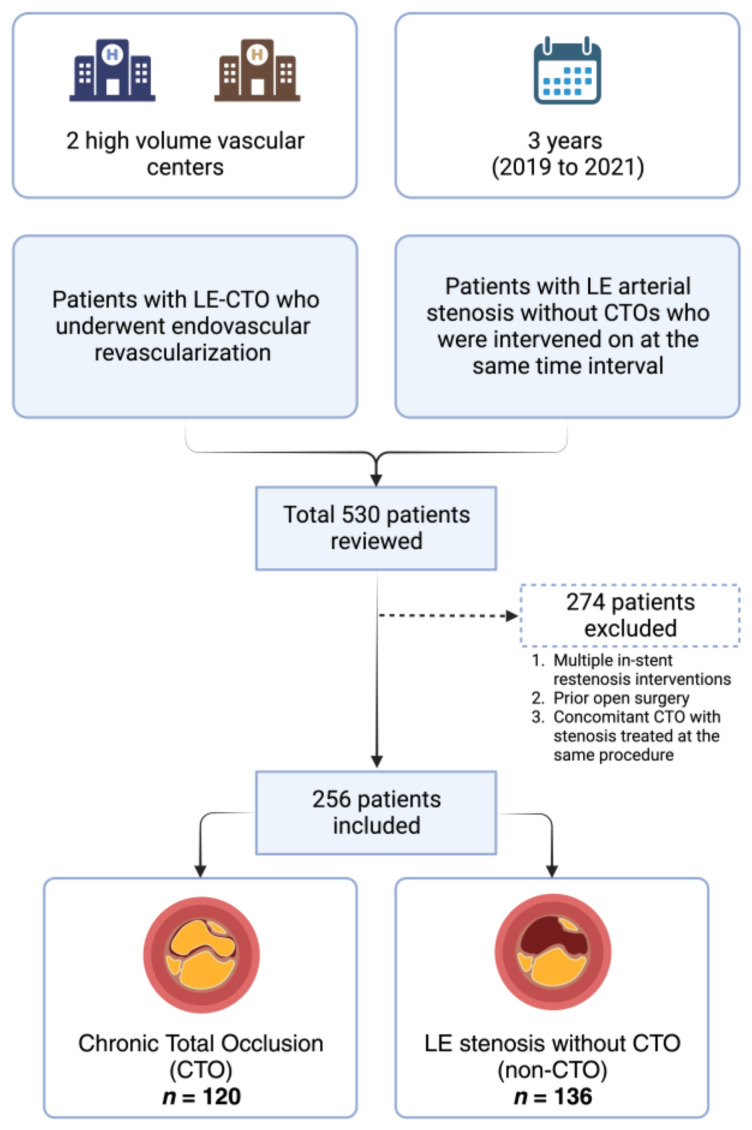
Study Flowchart.

Patients with LE-CTO who underwent endovascular revascularization during a 3-year period from 2019 to 2021 in two high-volume vascular centers were enrolled. Institutional Review Board approval was obtained with a waiver of informed consent because no intervention was performed and observational design. No additional interventions or variations from the usual care were performed. Baseline demographics, clinical, angiographic, and interventional characteristics were collected. An additional group of patients with LE arterial stenosis—who did not have CTOs—who were intervened on at the same time interval were used as a comparison group. The study flowchart is illustrated in Figure 1.

### 2.1. Study Population and Data Collection 

This research was conducted according to the Helsinki declaration. Waiver of informed consent was provided by the ethics committee of the enrolling institutions because this research project did not involve any variations from the normal clinical routine and protocol but just involved retrospective inclusion of patients for statistical analysis. All endovascular-treated LE-CTO lesions that were attempted to be crossed in the two centers within the pre-defined time interval were considered eligible for inclusion. Data extraction was performed by four different clinical data abstractors (experienced in the process of identifying and capturing key administrative and clinical data elements) using electronic medical records and angiograms from the two centers. Pertinent baseline demographic, clinical, laboratory values, angiographic, and procedural characteristics for all included patients were included in the analysis. 

### 2.2. Data Definitions and Outcomes 

Included patients were categorized based on the presence of a LE-CTO at the time of the intervention as evident from the angiogram review. Patients who had a CTO lesion but underwent an intervention for a non-CTO lesion were not included in the analysis (17 patients). Target lesion calcification was assessed based on angiogram review as none, mild, moderate, or severe and categorized subsequently into 2 groups (none/mild vs. moderate/severe). Peripheral Arterial Calcium Scoring System (PACSS) was used as a reference in defining the calcification grading system. In total, 120 patients with LE-CTO lesions were intervened on and 136 patients without LE-CTOs who were intervened for arterial stenosis were included in the two groups. There were no predetermined selection criteria for endovascular revascularization between the groups. Target lesion success was applied to treatments that achieved a residual stenosis of 30%, whereas procedure success was target lesion success without complications. Rutherford classification, which is routinely used in peripheral artery disease to categorize a patient based on their clinical and objective findings into 7 categories, was used in our study. Claudication was classified as Rutherford categories 1 to 3 and critical limb-threatening ischemia (CLTI) was classified as Rutherford categories 4 to 6. Smoking status was assessed at the time of the intervention and was separately charted for active and prior smoking. The age was analyzed in a continuous way but also in different age subgroups. Race was dichotomized into Caucasian, which was the single most common race encountered, and others. Body mass index (BMI) was handled similarly to age (both continuous and categorical comparisons). Medications of interest included statins, aspirin, and clopidogrel. Significant past medical problems included in the analysis were diseases such as diabetes mellitus (hemoglobin A1c > 6.4% and/or on treatment with anti-diabetic medications), congestive heart failure, abdominal aorta aneurysm, hypertension (blood pressure > 140/90 mmHg and/or on treatment with antihypertensive medications), hyperlipidemia, end-stage kidney disease (estimated glomerular filtration rate < 15 mL/min), coronary and carotid artery disease, prior myocardial infarction, prior stroke, chronic obstructive pulmonary disease, and history of any malignancy. The ankle-brachial index (ABI) and the toe-brachial index (TBI) were recorded at the time of the first presentation. The systolic blood pressure, hemoglobin A1c, and estimated glomerular filtration rate (eGFR) at the time of enrolment were also recorded. The TransAtlantic Inter-Society Consensus (TASC) classification of each one of the intervened lesions was recorded. Other angiographic and imaging characteristics for the data that were collected included computed tomography angiography (CT Angio) run-off, lesion length, thrombus, and restenosis status, type of procedure (single vs. multi-vessel intervention), fluoroscopy time, contrast volume used, crossing approach (anterograde vs. retrograde), access to site-related complications (hematoma, pseudoaneurysm) and procedure urgency. Interventional characteristics included in the analysis were the use of balloon angioplasty and cutting balloon, atherectomy device use, stenting, type of stent, stent maximum diameter, procedural success, complications (dissection perforation, other), aspirin or clopidogrel use post-procedure, and closure device use. 

### 2.3. Statistical Analysis

Continuous data were presented as median with interquartile range (IQR) and categorical data as absolute with relative frequencies. Continuous data were also expressed as means (presented in Supplementary Data). The normality of continuous variables was checked using the Shapiro–Wilk test (Supplementary Data). T-test was used for normally distributed data while Wilcoxon rank-sum (Mann–Whitney) was used for a comparison of the non-normally distributed data. The chi-square and Fisher’s exact tests were used for categorical variables. Logistic regression models were used to identify baseline variables associated with procedural success. The results of univariate logistic regression are given as the odds ratio (OR) with 95% confidence interval (CI). The threshold of statistical significance was *p* < 0.05. Multivariate regression analysis was performed on 2 models—model 1 for variables that were significant at 5% in univariate analysis, and model 2 with model 1 plus age and gender. All analyses were performed using STATA software (version 14.1; STATA Corporation, College Station, TX, USA).

## 3. Results

### 3.1. Patient Characteristics

A total of 256 patients with PAD who underwent LE endovascular revascularization procedures were included in the final analysis. Among them, 46.9% (*n* = 120) had CTO and 53.1% (*n* = 136) did not have CTO without any differences in mean age (67.5 vs. 65 years, *p* = 0.771). The baseline characteristics of the two groups are summarized in Table 1. 

The CTO group had an equal number of male and female patients, whereas in the non-CTO group, there was a male predominance (65.4%). Prior smoking history was significantly higher in the non-CTO group (92.7 vs. 70%, *p* < 0.001). The CTO group had a significantly higher prevalence of myocardial infarction history (29.2 vs. 18.26%, *p* = 0.05) and end-stage kidney disease (19.2 vs. 9.6%, *p* = 0.27), whereas the non-CTO group had a higher prevalence of dyslipidemia (84.6 vs. 69.8%, *p* = 0.005). A significantly higher proportion of CTO patients presented with CLTI (64.2 vs. 22.8%, *p* < 0.001), whereas the non-CTO group had a higher proportion of claudication (32.5 vs. 59.6%, *p* < 0.001). CTO patients had higher Rutherford classes (class 4–6) on presentation compared to non-CTO patients (*p* < 0.001). 

### 3.2. Angiographic Characteristics

Angiographic characteristics are presented in Table 2. TASC C and D lesions were more common in the CTO group. Both right and left leg CT Angio run-off vessels were smaller in the CTO group compared with the non-CTO group (right leg—means: 1.33 vs. 2.10 mm, medians: 1.0 vs. 2.0 mm, *p* < 0.001; left leg—means: 1.62 vs. 2.12 mm, medians: 2.0 vs. 2.0 mm, *p* = 0.003). Lesion length was significantly greater in the CTO group (means: 106.85 vs. 55.37 mm, medians: 100 vs. 40 mm, *p* < 0.001). Thrombotic lesions were less common in the non-CTO group (1.7 vs. 7.6%, *p* = 0.027), and restenosis cases were more prevalent in the non-CTO group (11.7 vs. 27.8%, *p* = 0.001). 

### 3.3. Procedural Characteristics

Procedural characteristics are summarized in Table 3. A significantly higher proportion of CTO patients underwent multi-vessel revascularization compared with non-CTO patients (72.5 vs. 53.7%; *p* = 0.002). CTO interventions had higher fluoroscopy time (means: 39.92 vs. 19.78 min, medians: 32 vs. 16.4 min, *p* < 0.001) without any significant difference in contrast volume used (means: 183.6 vs. 166.84 mL, medians: 179 vs. 150 mL, *p* = 0.159). A significant difference in procedural urgency was observed with a higher proportion of CTO patients undergoing urgent interventions (77 vs. 60%, *p* = 0.011). No differences were noted in the crossing approach and in the procedural complications. Procedural success was numerically more common in the non-CTO group (95.8 vs. 99.3%. *p* = 0.075), although the results did not meet statistical significance. There were two vessel perforation events in the CTO group vs. none in the non-CTO and eight dissections in the CTO group vs. 15 in the non-CTO. However, there were no statistically significant differences between the two groups. 

### 3.4. Differences among CTO Lesions Based on Successful vs. Unsuccessful Revascularization

Differences between CTO patients with and without successful revascularization are summarized in Table 4. Patients with ALI (4 patients) were excluded from regression analysis among the CTO patients. In the CTO group (*n* = 116), 87 patients (75%) had successful revascularization. There were no differences in success rates according to age, sex, race, or BMI. A significantly higher proportion of CTO patients with successful revascularization had a history of aspirin use (85 vs. 62%, *p* = 0.008). CTO patients with successful revascularization had a lower prevalence of malignancy (9 vs. 28%, *p* = 0.013). No differences were observed between the groups in terms of fluoroscopy time, contrast volume, crossing techniques, or access site complications. CTO patients with successful interventions had higher utilization of balloon PTA compared with those without successful intervention (91 vs. 76%, *p* = 0.038). CTO patients with successful revascularization had significantly lower complications compared with those without successful revascularization (0 vs. 33.3%, *p* < 0.001). There were eight patients with dissections and two with perforations among CTO patients without successful interventions.

### 3.5. Predictors of Successful Revascularization among Patients with Chronic Total Occlusion 

We performed univariate logistic regression analysis for all the baseline variables after excluding patients with ALI (four patients) to examine which of them had a significant association with successful endovascular revascularization (Table 5 and Supplementary Table S3). There was no significant association for sex, race, BMI, smoking status, hypertension, diabetes, concomitant coronary artery disease (CAD), and hyperlipidemia. Aspirin use (OR: 3.48; 95% CI: 1.33–9.07; *p* = 0.011) had a positive association with successful revascularization and prior malignancy (OR: 0.27; 95% CI: 0.09–0.8; *p* = 0.018) had a negative association with successful revascularization of LE CTO. These associations were still present in multivariate analysis (Supplementary Table S4).

## 4. Discussion

The aim of this study was to describe the baseline characteristics of PAD patients with and without CTO who underwent endovascular revascularization. We found that CTO patients had an equal sex distribution, higher severity of presentation, and underwent more urgent procedures that required intervening on multiple vessels and longer fluoroscopy time, among others. Additionally, we also described the predictors of a successful CTO revascularization. The use of aspirin and the absence of malignancy were associated with successful crossing. No angiographic predictors were identified in our analysis.

The coexistence of coronary and PAD is associated with higher adverse cardiovascular events and all-cause mortality [24,25]. Although CAD and PAD share many common risk factors, PAD was shown to increase the risk of cardiovascular death independently [26]. We noticed in our study that a higher proportion of PAD patients with CTOs had a history of previous MI as compared with those without CTOs. This is probably linked to the extensive progression of the atherosclerotic burden among this subset of PAD patients. In a study of 18,380 patients who underwent percutaneous coronary intervention (PCI), patients with PAD had significantly higher rates of a previous MI, whereas in the CHARISMA (Clopidogrel and Aspirin versus Aspirin Alone for the Prevention of Atherothrombotic Events) trial, 35% of the PAD patients had a history of previous MI [25,27]. Similarly, a higher proportion of end-stage kidney disease (ESKD) patients were found in the CTO group (19.2%). This is explained by the effect of ESKD on vessel wall calcium deposition and the more aggressive progression of the atherosclerotic process [28,29,30].

Another interesting finding in our study was that the prevalence of dyslipidemia was significantly lower among CTO patients compared with non-CTO, likely related to more aggressive treatment with lipid-lowering agents in patients with CTOs, who likely suffered also from extensive/earlier atherosclerotic disease. Although we did not find any significant differences in statin therapy rates between the two groups, this study did not examine the intensity, duration, and adherence to statin therapy. 

Gallagher et al. studied 413 CTO patients and observed that 304 (63.2%) presented with critical limb-threatening ischemia (CLTI) compared to 177 (36.8%) who presented with claudication [31]. Similarly, in our study, more CTO patients presented with CLTI, whereas non-CTO patients presented mostly with claudication prior to receiving an intervention. This was also reflected in the higher percentage of procedures classified as “urgent” in the CTO group but also by the higher percentage of multi-vessel interventions and longer fluoroscopy time in the CTO group compared with the non-CTO group. Although CTO is associated with severe advanced PAD, our study showed a significantly higher intervention for restenosis in the non-CTO group (28%) compared to the CTO group (12%). This can be explained by previously noted prevalence patterns of Tosaka I (focal non-occlusive) and Tosaka II (diffuse non-occlusive) restenosis lesions with a higher combined incidence compared to Tosaka III (occlusive) restenosis lesions [32,33,34]. CTOs have been shown in the past to be associated with more advanced diseases and worse outcomes [6,35]. These findings emphasize the severity of PAD associated with CTO lesions and the challenges posed to an endovascular interventionalist.

CTO lesions are difficult to cross because of the dense fibrous plaque that makes it challenging to advance the wire across the lesion. Over time, the plaque transforms and a combination of tissue aging, calcification, thrombus organization, and formation of dense fibrous caps on both ends of the lesion develops [15,36]. Despite the presence of multiple devices and techniques, the failure rates can still range between 15 and 30% [23,35,37]. The present study found a positive association between aspirin use and successful revascularization among the CTO group. This can be explained by the effects of aspirin on atheroma plaque progression. Ronaldo et al. demonstrated that aspirin is cardioprotective not only because of its antiplatelet effect but also because of its inhibition of cell proliferation and plaque growth, which is mediated by transforming growth factor-beta (TGF-beta) [38]. Mehta et al. studied cultured human coronary artery endothelial cells and demonstrated that aspirin has multiple cyclooxygenase-independent effects on the vascular endothelium [39]. Procedural success among CTO groups with aspirin use can be attributed to the inhibition of vascular smooth muscle cells, which makes crossing across a less muscular lesion easier. Similarly, a history of malignancy as a negative predictor of procedural success in CTO lesions can be attributed to the inflammatory state associated with malignancy. Atherosclerosis is an inflammatory state and an overlap of another pro-inflammatory condition such as malignancy increases the disease burden [40]. The absence of such a state can be compared with the anti-inflammatory and antioxidant effects of aspirin on these lesions [41,42]. However, this is the first mention of aspirin and the absence of malignancy as predictors of successful crossing in LE-CTO based on the literature review by the present authors. 

Previous studies tried to focus on specific vascular territories (coronaries, femoropopliteal, and infrapopliteal) and to provide specific angiographic variables that can be associated with successful crossing and crossing time [15,37,43,44]. In our analysis, we were unable to find any angiographic variables associated with successful crossing. Previous studies such as the CTOP (Chronic Total Occlusion Crossing approach based on plaque cap morphology) classification system tried to provide a guide to the crossing approach based on the plaque cap morphology and found that above-the-knee occlusive disease had better outcomes in terms of mortality and amputations compared with below-the-knee or multi-level lesions. [45] Unfortunately, we did not stratify our results based on the lesion location. Similarly, contrary to the previously published Infrapop-CTO score, we did not find calcification, lesion length, restenosis status, and stump morphology to affect crossing rates [43].

The management approach to CTO lesions is experiencing a shift towards endovascular interventions [35]. Despite the new devices and techniques such as drug-coated balloon angioplasty, re-entry devices, laser atherectomy, and anterograde approach, the baseline differences among the patients with CTO and non-CTO stenotic lesions have not been extensively studied [22]. Exploring the characteristic differences that predispose a patient with PAD to develop CTO lesions along with predictors of successful revascularization will provide further insights into managing these complex lesions successfully with minimally invasive techniques.

## 5. Limitations

This was a dual-center observational study, which was limited by the disadvantages related to non-randomized research. This study included a small sample size of 256 patients from only two centers, which may limit the generalizability and statistical power of the results. Obviously, the observational nature of the study was associated with its limitations. The study categorized patients based on ESKD status and did not categorize them based on all stages of chronic kidney disease. Although aspirin and clopidogrel were studied among preoperative medications, oral anticoagulants were not analyzed in our study. The study included patients presenting with ALI, which may not necessarily warrant the same decision making compared to other non-acute presentations. Furthermore, we categorized patients as ‘other’ for clinical presentation (17 patients in the non-CTO group). These patients could not be classified into ALI, CTLI, or claudication because some of the patients were diagnosed as part of surveillance testing and were not originally complaining of symptoms until further history was taken. In some other patients, it was not clear from the chart if they were Rutherford III or IV. Furthermore, lesion-specific data as per the TASC category were not studied in these patients. The study also did not analyze the effect of contrast exposure on the kidney function between the two groups and the change from baseline. The study did not categorize the interventions performed with drug-coated devices separately.

Additionally, the present study is limited by the fact that only endovascularly treated lesions were included. Some patients underwent multivessel intervention and there may be some limitations in identifying the culprit lesion in such patients. The lesion that was thought to be primarily responsible for symptoms was based on chart reviews and correlation with history, symptoms, and non-invasive testing anatomy when more than one vessel intervened. If there was one occlusion and one stenosis, lesions were excluded. The results might be different with the inclusion of surgically treated patients. All the included patients were treated by specific endovascular surgeons, which might limit the generalizability of our findings to other physicians and teams. This study did not evaluate the long-term follow-up data on the clinical outcomes and durability of the endovascular interventions. Despite these limitations, we think that our study benefits from its novel design and concept. 

## 6. Conclusions

While patients with CTO had more comorbidities and higher rates of urgent presentations for revascularization and underwent more complex procedures, the actual predictors of successful crossing of the occlusion did not seem to be related to the lesion characteristics themselves. Patients using aspirin at baseline had a favorable outcome, whereas those with a history of malignancy had an unfavorable outcome. Further studies are needed to compare the long-term outcomes of the two groups. 

## Figures and Tables

**Table 1:** Baseline characteristics between CTO vs. non-CTO groups.

	CTO (*n* = 120)	Non-CTO (*n* = 136)	*p*
Sex, male	60/120 (50%)	89/136 (65%)	0.012
Age, years, median (IQR)	65.0 (56.0–75.5)	67.5 (58.5–75.0)	0.771
Race			0.109
Caucasian	48/120 (40%)	68/136 (50%)	
Other	72/120 (60%)	68/136 (50%)	
Median BMI, kg/m^2^ (IQR)	23.2 (19.3–26.4)	23.3 (20.6–26.8)	0.389
Current smoking	30/120 (25%)	49/136 (36%)	0.057
Past smoking	84/120 (70%)	126/136 (93%)	<0.001
Statin	85/108 (79%)	109/131 (83%)	0.376
Aspirin	96/120 (80%)	117/135 (87%)	0.152
Clopidogrel	51/69 (74%)	58/69 (84%)	0.144
Diabetes mellitus	70/119 (59%)	67/136 (49%)	0.127
CHF	32/119 (26%)	25/136 (18%)	0.104
Hypertension	101/120 (84%)	124/136 (91%)	0.086
CAD	58/120 (48%)	67/136 (49%)	0.882
Dyslipidaemia	83/120 (70%)	115/135 (85%)	0.005
Stroke/TIA	24/120 (20%)	18/136 (13%)	0.145
MI	33/113 (29%)	21/115 (18%)	0.05
ESKD (eGFR < 15 mL/min)	23/120 (19%)	13/136 (10%)	0.027
Presentation			<0.001
ALI	4/120 (3%)	7/136 (5%)	
CLTI	77/120 (64%)	31/136 (23%)	
Claudication	39/120 (33%)	81/136 (60%)	
Other	0/120 (0%)	17/136 (13%)	

Abbreviations: AAA, abdominal aortic aneurysm; ALI, acute limb ischemia; BMI, body mass index; CAD, coronary artery disease; CHF, congestive heart failure; CLTI, critical limb-threatening ischemia; COPD, chronic obstructive pulmonary disease; CTO, chronic total occlusion; eGFR: estimated glomerular filtration rate; ESKD, end-stage kidney disease; IQR, inter-quartile range; MI, myocardial infarction; TIA, transient ischemic attack.

**Table 2:** Angiographic characteristics between CTO vs. non-CTO groups.

	CTO (*n* = 120)	Non-CTO (*n* = 136)	*p*
TASC Classification			<0.001
TASC A	10/105 (10%)	49/112 (44%)	
TASC B	27/105 (26%)	41/112 (37%)	
TASC C	37/105 (35%)	14/112 (13%)	
TASC D	31/105 (30%)	8/112 (7%)	
Calcification			0.353
Moderate to severe	56/119 (47%)	54/131 (41%)	
None to mild	63/119 (53%)	77/131 (59%)	
Thrombotic lesion	2/120 (2%)	10/131 (8%)	0.027
Restenosis intervention	14/120 (12%)	37/133 (28%)	0.001

Abbreviations: CTO, chronic total occlusion; TASC, Trans-Atlantic Inter-Society Consensus.

**Table 3:** Procedural characteristics between CTO vs. non-CTO groups.

	CTO (*n* = 120)	Non-CTO (*n* = 136)	*p*
Multi Vessel Intervention	87/120 (73%)	73/136 (54%)	0.002
Fluoroscopy Time, min, median (IQR)	32.9 (21.3–45.0)	16.4 (10.2–26.5)	<0.001
Contrast Volume, ml, median (IQR)	178.5 (137.5–240.0)	150.0 (100.0–220.0)	0.159
Access Site			0.381
Anterograde	13/117 (11%)	20/133 (15%)	
Retrograde Pedal	1/117 (1%)	0/133 (0%)	
Retrograde	103/117 (88%)	113/133 (85%)	
Access Site Complication			0.541
None	117/120 (98%)	133/136 (98%)	
Haematoma	2/120 (2%)	3/136 (2%)	
Pseudoaneurysm	1/120 (1%)	0/136 (0%)	
Procedure Urgency			0.011
Elective	22/120 (18%)	47/136 (35%)	
Emergent	6/120 (5%)	8/136 (6%)	
Urgent	92/120 (77%)	81/136 (60%)	
Successful revascularization	115/120 (96%)	132/133 (99%)	0.075
Complication			0.196
None	110/120 (92%)	117/133 (88%)	
Dissection	8/120 (7%)	15/133 (11%)	
Perforation	2/120 (2%)	0/133 (0%)	
Other	0/120 (0%)	1/133 (1%)	

Abbreviations: CTO, chronic total occlusion; IQR, inter-quartile range.

**Table 4:** Differences between CTO patients with and without successful endovascular revascularization.

	Successful Revascularization (*n* = 87)	Unsuccessful Revascularization (*n* = 29)	*p*
Gender, male	40 (46%)	17 (59%)	0.238
Age, years, median (IQR)	67.0 (55.0–74.0)	64.0 (58.0–76.0)	0.526
Caucasian Race	38 (44%)	9 (31%)	0.230
Median BMI, kg/m^2^ (IQR)	23.2 (19.3–26.6)	23.2 (21.0–25.8)	0.729
Current smoking	22 (25%)	5 (17%)	0.375
Past smoking	60 (69%)	20 (69%)	1.000
Statin	62 (79%)	22 (81%)	0.823
Aspirin	74 (85%)	18 (62%)	0.008
Clopidogrel	36 (73%)	14 (74%)	0.986
Diabetes mellitus	47 (55%)	21 (72%)	0.092
CHF	25 (29%)	7 (24%)	0.608
Hypertension	70 (80%)	27 (93%)	0.151 *
CAD	43 (49%)	14 (48%)	0.915
Dyslipidaemia	60 (69%)	21 (75%)	0.543
Malignancy	8 (9%)	8 (28%)	0.013
Stroke/TIA	18 (21%)	5 (17%)	0.687
MI	26 (32%)	5 (18%)	0.150
ESKD (eGFR < 15 mL/min)	16 (18%)	6 (21%)	0.784
Presentation			0.427
CLTI	56 (64%)	21 (72%)	
Claudication	31 (36%)	8 (28%)	
TASC Classification			0.138 *
TASC A	6 (8%)	4 (17%)	
TASC B	21 (27%)	6 (26%)	
TASC C	31 (39%)	4 (17%)	
TASC D	21 (27%)	9 (39%)	
Calcification			0.481
Moderate to severe	38 (44%)	15 (52%)	
None to mild	48 (56%)	14 (48%)	
Thrombotic lesion	1 (1%)	1 (3%)	0.439 *
Restenosis intervention	10 (11%)	2 (7%)	0.728 *
Multi Vessel Intervention	63 (72%)	20 (69%)	0.722
Fluoroscopy Time, min, median (IQR)	29.6 (19.2–42.0)	39.5 (31.6–49.8)	0.053
Contrast Volume, ml, median (IQR)	180.0 (140.0–240.0)	163.5 (130.0–227.5)	0.705
Balloon PTA	79 (91%)	22 (76%)	0.038
Access Site			1.000
Anterograde	10 (12%)	3 (10%)	
Retrograde Pedal	1 (1%)	0 (0%)	
Retrograde	74 (87%)	26 (90%)	
Access Site Complication			0.061 *
None	0 (0%)	2 (7%)	
Hematoma	86 (99%)	27 (93%)	
Pseudoaneurysm	1 (1%)	0 (0%)	
Procedure Urgency			0.179 *
Elective	18 (21%)	4 (14%)	
Emergent	1 (1%)	2 (7%)	
Urgent	68 (78%)	23 (79%)	
Complication			<0.001
None	0 (0%)	8 (28%)	
Dissection	87 (100%)	19 (66%)	
Perforation	0 (0%)	2 (7%)	

Abbreviations: AAA, abdominal aortic aneurysm; ALI, acute limb ischemia; BMI, body mass index; CAD, coronary artery disease; CHF, congestive heart failure; CLTI, critical limb-threatening ischemia; COPD, chronic obstructive pulmonary disease; CTO, chronic total occlusion; eGFR: estimated glomerular filtration rate; ESKD, end-stage kidney disease; IQR, inter-quartile range; MI, myocardial infarction; PTA, percutaneous transluminal angioplasty; SBP, systolic blood pressure; TASC, Trans-Atlantic Inter-Society Consensus; TIA, transient ischemic attack. * Fischer’s test.

**Table 5:** Univariate analysis of dependent variables among patients with LE-CTO lesions predicting procedural success.

Dependent Variable	Odds Ratio (95% CI)	*p*
Aspirin vs. No aspirin	3.48 (1.33–9.07)	**0.011**
Malignancy vs. No Malignancy	0.27 (0.09–0.80)	**0.018**

Abbreviations: CI, Confidence interval.

## Data Availability

The data presented in this study are available on request from the corresponding author. The data are not publicly available due to ethical restrictions.

## Supplementary Materials

The following supporting information can be downloaded at: https://www.mdpi.com/article/10.3390/medicina59112029/s1. Table S1. Continuous variables in Tables 1–3, presented as means; Table S2. Continuous variables in Table 4, presented as means; Table S3. Multivariate regression models. Table S4. Univariate analysis of dependent variables among patients with LE-CTO lesions pre-dicting procedural success.
