# Supplementary material for: Differences between Lower Extremity Arterial Occlusion vs. Stenosis and Predictors of Successful Endovascular Interventions
Source: Medicina (Kaunas). 2023 Nov 17;59(11):2029. doi: 10.3390/medicina59112029 (PMC10673017; doi:10.3390/medicina59112029)
Supplement: Supplementary file 1 [file medicina-59-02029-s001.zip › medicina-2601223-supplementary.pdf]

Table S1. Continuous variables in Tables 1–3, presented as means.

Table	Continuous Variable	Shapiro-Wilk Test (normality test)	Significance test	CTO group		Non-CTO group		P - value
				Mean	SD	Mean	SD	
Table 1	Age	0.015	Wilcoxon rank-sum (Mann–Whitney) test	65.60	12.48	66.04	11.97	0.687
Table 1	BMI	0.045	Wilcoxon rank-sum (Mann–Whitney) test	23.18	5.28	23.72	4.57	0.441
Table 2	Right Angio Runoff	0.865	t-test	1.33	1.10	2.10	0.94	<0.001
Table 2	Left Angio Runoff	0.433	t-test	1.62	0.97	2.12	0.76	0.003
Table 2	Lesion length, mm	<0.001	Wilcoxon rank-sum (Mann–Whitney) test	106.85	68.73	55.37	45.29	<0.001
Table 3	Fluoroscopy time, min	<0.001	Wilcoxon rank-sum (Mann–Whitney) test	39.92	40.13	19.78	13.68	<0.001
Table 3	Contrast Volume, ml	<0.001	Wilcoxon rank-sum (Mann–Whitney) test	183.60	70.96	166.84	95.81	0.035

Table S2. Continuous variables in Table 4, presented as means.

Table	Continuous Variable	Shapiro-Wilk Test (normality test)	Significance test	Successful revascularization among CTO group		Unsuccessful revascularization among CTO group		P - value
				Mean	SD	Mean	SD	
Table 4	Age	0.27859	t-test	65.31	12.45	66.47	12.74	0.662
Table 4	BMI	0.21139	t-test	23.01	5.37	23.67	5.06	0.563
Table 4	Right Angio Runoff	0.95244	t-test	1.38	1.06	1.18	1.25	0.607
Table 4	Left Angio Runoff	1.00000	t-test	1.72	0.94	1.31	1.03	0.191
Table 4	Lesion length, mm	<0.001	Wilcoxon rank-sum (Mann–Whitney) test	111.61	71.13	91.00	58.46	0.155
Table 4	Fluoroscopy time, min	<0.001	Wilcoxon rank-sum (Mann–Whitney) test	34.07	21.28	52.01	62.31	0.013
Table 4	Contrast Volume, ml	0.13425	t-test	185.02	69.47	180.52	75.26	0.779

Table S3. Multivariate regression models.

	Model 1		Model 2	
**VARIABLES**	**HR (CI 95%)**	**P-value**	**HR (CI 95%)**	**P-value**
Male	-	-	0.58 (0.24 - 1.42)	0.234
Age	-	-	1.00 (0.96 - 1.04)	964
Aspirin	3.21 (1.23 - 8.37)	0.017	3.15 (1.16 - 8.60)	0.025
Malignancy	0.29 (0.10 - 0.87)	0.027	0.29 (0.09 - 0.88)	0.29
Constant	1.52 (0.66 - 3.52)	0.328	2.17 (0.15 - 30.75)	0.568
Observations	120		120	
Wald	10.86		11.57	
Pseudo R2	0.0801		0.0908	

Model 1: significant variables at 5% in univariate analysis
Model 2: significant variables at 5% in univariate analysis plus gender and age

Table S4. Univariate analysis of dependent variables among patients with LE-CTO lesions predicting procedural success.

Dependent variable	Odds ratio (95% CI)	p
Male vs female sex	0.58 (0.25 - 1.36)	0.210
Age	0.99 (0.96 - 1.03)	0.661
Age categories	1.03 (0.57 - 1.85)	0.933
Caucasian race vs other races	1.78 (0.73 - 4.34)	0.202
BMI	0.98 (0.91 - 1.05)	0.539
BMI categories	0.85 (0.48 - 1.51)	0.574
Current smoking vs No current smoking	1.45 (0.53 - 4.01)	0.469
Past smoking vs No past smoking	1.00 (0.40 - 2.47)	1.000
Statin vs No statin	1.01 (0.35 - 2.90)	0.984
Aspirin vs No aspirin	3.43 (1.32 - 8.88)	0.011
Clopidogrel vs No clopidogrel	1.02 (0.30 - 3.41)	0.979
Diabetes mellitus vs No diabetes mellitus	0.53 (0.22 - 1.28)	0.156
CHF vs No CHF	1.28 (0.49 - 3.38)	0.613
Hypertension vs No hypertension	0.31 (0.07 - 1.42)	0.131
AAA vs No AAA	1.35 (0.14 - 12.68)	0.079
Carotid Artery Stenosis vs Carotid Artery Stenosis	2.93 (0.63 - 13.72)	0.172
CAD vs No CAD	1.09 (0.48 - 2.51)	0.834
COPD vs No COPD	1.20 (0.36 - 3.98)	0.769
Dyslipidaemia vs No Dyslipidaemia	0.84 (0.33 - 2.14)	0.721
Malignancy vs No Malignancy	0.27 (0.09 - 0.80)	0.018
Stroke/TIA vs No Stroke/TIA	1.34 (0.45 - 3.98)	0.601
MI vs No MI	2.40 (0.82 - 6.99)	0.109

ESKD (eGFR <15 ml/min) vs No ESKD	0.93 (0.33 - 2.64)	0.894
First visit SBP	0.98 (0.96 - 1.00)	0.067
HbA1c at time of 1st visit	1.75 (0.99 - 3.08)	0.053
GFR	1.00 (0.99 - 1.01)	0.837
Presentation		
CLTI vs No CLTI	0.71 (0.29 - 1.72)	0.445
Claudication vs No Claudication	1.44 (0.57 - 3.63)	0.434
Rutherford classification	0.83 (0.59 - 1.17)	0.287
TASC classification		
TASC A	0.40 (0.10 - 1.57)	0.188
TASC B	1.05 (0.37 - 3.01)	0.928
TASC C	2.48 (0.84 - 7.35)	0.101
TASC D	0.62 (0.24 - 1.63)	0.334
Moderate-to-severe calcification	0.71 (0.31 - 1.64)	0.429
Lesion length	1.01 (1.00 - 1.01)	0.188
Thrombotic lesion	0.33 (0.02 - 5.44)	0.435
Restenosis intervention	2.15 (0.45 - 10.30)	0.336
Multi-vessel intervention	1.18 (0.47 - 2.94)	0.725
Fluoroscopy time	0.98 (0.96 - 1.00)	0.101
Contrast volume	1.00 (0.99 - 1.01)	0.785
Access site		
Anterograde	1.17 (0.30 - 4.59)	0.823
Retrograde	0.77 (0.20 - 2.98)	0.702
Procedure urgency		
Elective	1.63 (0.50 - 5.27)	0.419
Emergent	0.65 (0.11 - 3.78)	0.632

Urgent	0.77 (0.28 - 2.14)	0.620
